# The *Physalis peruviana* leaf transcriptome: assembly, annotation and gene model prediction

**DOI:** 10.1186/1471-2164-13-151

**Published:** 2012-04-25

**Authors:** Gina A Garzón-Martínez, Z Iris Zhu, David Landsman, Luz S Barrero, Leonardo Mariño-Ramírez

**Affiliations:** 1 Plant Molecular Genetics Laboratory, Center of Biotechnology and Bioindustry (CBB), Colombian Corporation for Agricultural Research (CORPOICA), Bogota, Colombia; 2 Computational Biology Branch, National Center for Biotechnology Information, National Library of Medicine, National Institutes of Health, United States of America, Bethesda, MD, USA; 3 PanAmerican Bioinformatics Institute, Santa Marta, Magdalena, Colombia

**Keywords:** *P. peruviana*, Solanaceae, ESTs, Functional annotation, Gene model, Phylogenetics

## Abstract

**Background:**

*Physalis peruviana* commonly known as Cape gooseberry is a member of the Solanaceae family that has an increasing popularity due to its nutritional and medicinal values. A broad range of genomic tools is available for other Solanaceae, including tomato and potato. However, limited genomic resources are currently available for Cape gooseberry.

**Results:**

We report the generation of a total of 652,614 *P. peruviana* Expressed Sequence Tags (ESTs), using 454 GS FLX Titanium technology. ESTs, with an average length of 371 bp, were obtained from a normalized leaf cDNA library prepared using a Colombian commercial variety. *De novo* assembling was performed to generate a collection of 24,014 isotigs and 110,921 singletons, with an average length of 1,638 bp and 354 bp, respectively. Functional annotation was performed using NCBI’s BLAST tools and Blast2GO, which identified putative functions for 21,191 assembled sequences, including gene families involved in all the major biological processes and molecular functions as well as defense response and amino acid metabolism pathways. Gene model predictions in *P. peruviana* were obtained by using the genomes of *Solanum lycopersicum* (tomato) and *Solanum tuberosum* (potato). We predict 9,436 *P. peruviana* sequences with multiple-exon models and conserved intron positions with respect to the potato and tomato genomes. Additionally, to study species diversity we developed 5,971 SSR markers from assembled ESTs.

**Conclusions:**

We present the first comprehensive analysis of the *Physalis peruviana* leaf transcriptome, which will provide valuable resources for development of genetic tools in the species. Assembled transcripts with gene models could serve as potential candidates for marker discovery with a variety of applications including: functional diversity, conservation and improvement to increase productivity and fruit quality. *P. peruviana* was estimated to be phylogenetically branched out before the divergence of five other Solanaceae family members, *S. lycopersicum*, *S. tuberosum*, *Capsicum spp*, *S. melongena* and *Petunia spp*.

## Background

*Physalis peruviana*, also known as Cape gooseberry is a tropical fruit from the Solanaceae family, which includes many agriculturally important crops including potato, tomato, pepper, eggplant and tobacco [1]. The Cape gooseberry fruit contains high levels of vitamin A, C and B-complex, as well as compounds of anti-inflammatory and antioxidant properties [2]. Supercritical carbon dioxide extracts of *P. peruviana* leaves were shown to induce cell cycle arrest and apoptosis in human lung cancer H661 cells [3]. Recently, 4β-Hydroxywithanolide (4βHWE) isolated from *P. peruviana* aerial parts (stems and leaves) was demonstrated to be a potential DNA-damaging and chemotherapeutic agent against lung cancer [4]. In Colombia, this fruit has become promissory with high demand in European markets, mainly due to its unique taste, attractive color and shape as well as its potential health value. *P. peruviana* is a source of health related compounds found in the fruit and other parts of the plant including leaves and steams. Despite its nutritional and medical importance, current absence of *P. peruviana* genetic and genomic resources makes in-depth molecular studies on the plant difficult. Until this study, there were only a few partial *P. peruviana* gene sequences in public databases, mainly as a result of phylogenetic studies in the Solanaceae family [5,6]. Therefore, there is a pressing need for efforts to obtain global genetic and genomic information from the Cape gooseberry, *P. peruviana*.

Advances in next generation sequencing (NGS) technology over the past few years have made it possible to rapidly perform *de novo* transcriptome and even genome assembly for non-model organisms with no or little prior genomic data available [7] . However, polyploidy and the large size of many plant genomes, which is predominantly due to amplification of repetitive elements or sometimes partial genome duplication [8], pose challenges to *de novo* whole genome assembly of plants. As such, EST sequencing, which avoids non-coding and repetitive DNA components, is a cost-effective and commonly used strategy to analyze the transcribed portion of a genome. Availability of ESTs represent a valuable resource for research as they provide comprehensive information regarding the transcriptome facilitating gene discovery and genome annotation and aiding in the determination of phylogenetic relationships [9]. An increasing number of successful studies have been published describing EST sequencing and *de novo* transcriptome assembly for large-scale gene discovery [9-18].

**Figure 1:**
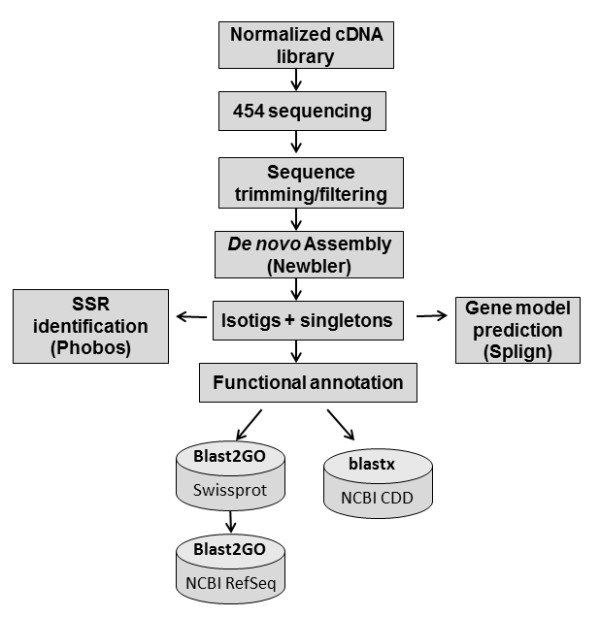
Schematic representation of the overall sequencing and annotation workflow for the Cape gooseberry transcriptome.

Here we describe the sequencing and assembly of the first *P. peruviana* leaf transcriptome from its cDNA-derived ESTs using the 454 GS-FLX Titanium technology, as well as *in silico* functional annotation and gene model prediction of the assembled transcriptome. The overall workflow of the project is represented in Figure 1. This first transcriptome draft will provide valuable resources for the development of molecular genetic tools that can be used in agronomic trait related marker discoveries, in addition to studies that aim to solve phytosanitary, fruit quality and production problems.

## Results and discussion

### EST sequencing and assembly

**Figure 2:**
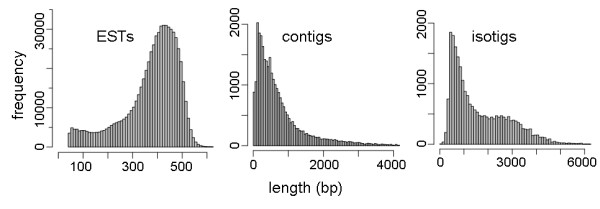
**Length distributions of *****P. peruviana***** EST reads (left), assembled contigs (center) and isotigs (right).** Data obtained after sequencing with three fourths run of 454 GS FLX Titanium of the normalized leaf cDNA library.

We performed three fourths 454 GS FLX Titanium run on one normalized cDNA library constructed from *P. peruviana* leaf tissue, generating approximately 336 Mbp of sequence data from 652,614 reads with an average length of 375 bp (Figure 2). After a trimming process by SeqClean [19], which removes adaptors, primer sequences, poly-A tails, as well as short, longer and low quality sequences, a total of 641,512 high quality reads were obtained with an average length of 371 bp. *De novo* transcriptome assembly was performed using Newbler 2.5.3 [20], which has been shown to perform better than a number of other commonly used assemblers [21]. Table 1 shows the transcriptome sequencing and assembly statistics, 79.66% of the reads were assembled into 29,911 contigs, and then further into 24,014 isotigs, with an average assembled length of 1,638 bp. The isotig N50 length was 2,504 bp. All isotigs that share common contigs belong to the same isogroup, presumably equivalent to one gene locus containing multiple alternatively spliced transcripts. The 24,014 assembled isotigs are part of 14,049 isogroups (equivalent to an average 1.7 transcripts per gene), among which 9,655 isogroups have only one isotig each. Isotigs whose length exceeded 200 bp (23,964 in total) were kept for further analysis. The remaining 20.34% reads are singletons, which cannot be connected with any other reads. The 110,921 singletons were kept for further analysis. The average coverage of assembled isotigs is estimated to be 9.1X. The number goes down to 3.9X if we include all the singletons as the effective transcribed portion. Isotigs and singletons together will be referred as cDNAs in the rest the manuscript. The raw data files are available at the National Center for Biotechnology Information (NCBI) Sequence Read Archive (SRA) accession number SRP005904. The assembled reads were deposited in the Transcriptome Shotgun Assembly (TSA) Database [GenBank:JO124085-JO157957].

**Table 1:** *P. peruviana* transcriptome assembly overview

	**Filtered EST reads**	**Contigs**	**Isotigs**	**Isogroups**	**Singletons**
	641,512	29,911	24,014	14,049	110,921
**Average length (bp)**	371	743	1,638		354
**N50 size (bp)**		1,438	2,504		

### Functional annotation

As the first step for assigning putative functions to the *P. peruviana* transcriptome, BLASTX searches [22,23] were used to align the cDNAs to the UniProtKB/Swiss-Prot and NCBI RefSeq databases. A total of 19,162 isotigs and 35,428 singletons had a BLAST hit (with an expectation value < 1e-5) to known proteins and matched 8,721 and 15,192 unique protein accessions, respectively. More than 99% of the BLASTX hits from both isotigs and singletons were from plant proteins. Compared to isotigs, a much greater percentage of singletons do not have any significant hits (68%), which could be mainly due to their short lengths. Using Blast2GO [24], we retrieved gene ontology (GO) terms and enzyme commission numbers (EC) for the *P. peruviana* cDNAs (Table 2) from the BLASTX output described above. A total of 33,105 GO terms were assigned to 12,672 cDNAs (including isotigs and singletons). Among all the GO terms extracted, 13,935 (42%) belong to the Molecular Function class, 10,375 (31%) to Biological Process class and 8,795 (27%) to Cellular Component class. There are 7,519 cDNAs assigned to multiple GO terms.

**Table 2:** *P. peruviana* transcriptome functional annotation overview

	**Isotigs**	**Singletons**	**Total**
Sequences with BLAST hits	19,162	35,428	54,590
Sequences annotated with GO terms	4,915	7,757	12,672
GO Terms associated with the sequences	12,675	20,430	33,105
Sequences associated with EC numbers	601	1,070	1,671

**Figure 3:**
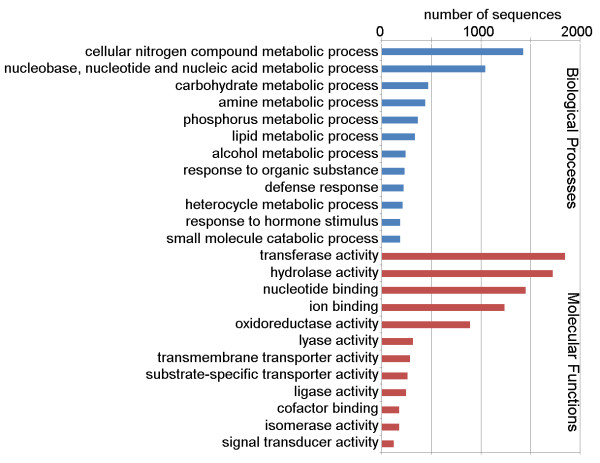
**Gene Ontology (GO) distributions for the Cape gooseberry transcriptome.** Main functional categories in the biological process (BP, level four) and molecular function (MF, level three) found in the transcriptome relevant to plant physiology. Results are from Blast2GO analysis.

The biological process (BP) GO category comprise different types of metabolic processes which are the most represented categories: there are 4,620 sequences associated with metabolic processes (GO level 2), which is expected, since the metabolic network in plants is by far more extensive compared to other organisms [25]. We found GO terms associated with primary metabolites, which include the universal building blocks of sugars, amino acids, nucleotides, lipids, and energy sources that are essential for plant survival. Additionally, we found GO terms associated with secondary metabolites that play key roles in maintaining plant fitness including ones that function in the protection of plants against microbial, viral infections and UV radiation. Shown in Figure 3 are a number of GO terms (BP category, level 4) that are abundant and relevant to plant physiology, like the metabolic processes of nitrogen compound, nucleotide, carbohydrate, amine and phosphorus. Another category worthy to mention is “response to stimulus” (BP category, level 2). We found 1,120 sequences associated with this category, which include candidate genes for resistance to pathogen attacks. Shown in Figure 3 are a number of level 4 GO categories including: response to organic substance, defense response and response to hormone stimulus. In the molecular function (MF) category, 30% of the *P. peruviana* cDNAs have high similarity to proteins with transferase or hydrolase activity (GO level 3) that includes genes associated with secondary metabolic synthesis pathways [9,10]. Other abundant level 3 MF categories include: nucleotide binding, ion binding and oxidoreductase binding (Figure 3).

We were able to assign 129 unique enzyme commission (EC) numbers to 1,671 *P. peruviana* cDNAs, where 25 unique EC numbers were in turn assigned to 52 metabolic pathways linked to 1,255 cDNAs (Table 3). We found 187 cDNAs involved in thiamine metabolism in addition to 84 sequences associated with secondary metabolite biosynthesis and 53 assigned to the phenylpropanoid biosynthesis pathway. These pathways are of particular interest in *Physalis* as thiamine has been known to induce defense response in plants through the salicylic acid and Ca^2+^-related signaling pathways [26,27] and may play roles in biotic or abiotic stress [28]. Furthermore, secondary metabolites such as phenylpropanoids play important roles in resistance mechanisms to pathogens and recently have also been used in medicinal applications including antioxidants, anticancer and anti-inflammatories [2,28].

**Table 3:** Main metabolic pathways associated to *P. peruviana* transcripts

KEGG* metabolic pathways	**Number of transcripts**
General metabolic pathways	301
Purine metabolism	193
Thiamine metabolism	187
Biosynthesis of secondary metabolites	84
Biosynthesis of phenylpropanoids	53
Drug metabolism - other enzymes	48
Oxidative phosphorylation	44
Tropane, piperidine and pyridine alkaloid biosynthesis	42
Phenylalanine metabolism	25
Biosynthesis of plant hormones	14
Biosynthesis of alkaloids derived from terpenoid/polyketide	14
Biosynthesis of terpenoids and steroids	14
Other pathways	236

* KEGG: Kyoto Encyclopedia of Genes and Genomes [29].

### Protein domains encoded by the *P. Peruviana* leaf transcriptome

A total of 12,974 *P. peruviana* cDNAs were found to have significant similarities to 3,117 protein domains present in the NCBI CDD (Conserved Domain Database) [30]. The most abundant domain present in proteins encoded by the *P. peruviana* transcriptome is the pentatricopeptide repeat domain (PPR), found in 350 cDNAs. The PPR containing proteins are commonly found in the plant kingdom and although its function is still unclear, the PPR domain has been found in proteins involved in RNA editing in a number of recent studies [31-34]. Following the PPR domain, the next three most commonly found domains in the *P. peruviana* transcriptome are: protein kinase domain (294 cDNAs), NB-ARC domain (190 cDNAs) and WD40 domain (123 cDNAs). Protein kinases are one of the largest protein families in plants, involved in a wide variety of physiological processes [35], like calcium-dependent protein kinases and MAP kinases which are involved in the recognition of elicitors or pathogens and the subsequent activation of defense response in plants [36]. The NB-ARC domain is a nucleotide-binding motif shared by plant resistant gene products involved in regulated cell death [37,38]. The WD40 domain, whose common function is coordinating multi-protein complex assemblies, is found in a large number of eukaryotic proteins that cover a wide variety of functions including adaptor and regulatory modules in signal transduction, pre-mRNA processing and cytoskeleton assembly [39,40]. Additionally, the WD40 domain is critically involved in the ubiquitin proteasome pathway which regulates photomorphogenesis, flowering and abiotic stress response in plants [41].

Other frequently found domains include: RNA recognition motif (115 cDNAs), RING-finger domain (96 cDNAs), Leucine rich repeat N-terminal domain (89 cDNAs), tyrosine kinase catalytic domain (84 cDNAs), all of which are commonly found in eukaryotic cells and involved in a broad range of biological processes. The data is summarized in Table 4.

**Table 4:** Protein domains identified in *P. peruviana* transcriptome

CDD* Identifier	**Domain name**	**Number of cDNAs**
193328, 188079	Pentatricopeptide repeat domain (PPR motif)	350
173623, 189373	Protein kinase domain	294
144508	NB-ARC domain	190
29257	WD40 domain	123
128654	RNA recognition motif	115
29102	RING-finger domain	96
191981	Leucine rich repeat N-terminal domain	89
128515	Tyrosine kinase catalytic domain	84
	Others	11633

*CDD: Conserved Domain Database.

Out of the 110,921 singletons, there are 9,909 of them (length >200 bp) where GO term(s) were assigned to the sequence through Blast2GO (see Materials) or where a significant similarity to a well-characterized protein domain from NCBI CDD was found. We deposited the 9,909 singletons described above, in addition to the 24,024 assembled isotigs in the NCBI’s TSA (Transcriptome Shotgun Assembly) sequence database, which is available at GenBank (accessions JO124085-JO157957). Those sequences with their functional annotations, including GO terms and domain similarity related description, are also provided as Additional file 1: ‘Cape gooseberry cDNAs’.

### *In silico* SSR marker identification

The presence of Simple Sequence Repeats (SSRs) in the *P. peruviana* transcriptome was identified *in silico* using Phobos [42]. A total of 5,971 SSR loci were found in the Cape gooseberry cDNAs, where imperfect motifs were the most abundant (5,568), in contrast to 403 loci representing perfect motifs (Table 5). Microsatellites were searched in cDNAs avoiding redundant results in isotigs, considering that searching in the alternative transcripts could lead us to predict the same SSRs in different isotigs corresponding to the same isogroup. Trinucleotide (1,068) and hexanucleotide (3,036) motifs were the most commonly found repetitions in the *P. peruviana* leaf transcriptome, accounting for 68% of the SSRs identified, in contrast to other plant studies where tri- and dinucleotides were the most commonly found repeat units [18,43,44].

**Table 5:** SSRs identified in *P. peruviana* cDNAs

**Motif**	**PERFECT**	**IMPERFECT**
Dinucleotide	69	275
Trinucleotide	150	918
Tetranucleotide	28	465
Pentanucleotide	22	1,008
Hexanucleotide	134	2,902
**TOTAL**	403	5,568

We recently reported the first set of microsatellite markers developed for *P. peruviana* and related species [45] where the large majority of SSR loci was found in untranslated regions (UTRs) of transcripts with similarity to known proteins in public databases, leading to the identification of two novel polymorphic SSRs related to proteins involved in pathogen defense response. SSRs prioritization for plant breeding programs can be done via functional annotation of cDNAs associated with predicted SSRs and Gene Ontology annotations like ones involved in plant defense. Here we used and updated functional annotation of the transcriptome and the entire collection of assembled transcripts to report ten novel predictions for cDNA-derived SSRs in Cape gooseberry. These SSRs are associated with proteins with gene ontology annotations involved in plant defense to biotic stress such as defense response to fungus, programmed cell death, callose deposition in cell wall during defense response, plant hypersensitive response, and jasmonic acid, ethylene and salicylic acid hormones ( Additional file 2: ‘Functional annotation of ten *Physalis peruviana* SSRs markers related to plant defense’). The SRRs obtained in this study are the raw materials for future studies in genetic variation among *Physalis* populations, which can be used for: construction of genetic maps, quantitative trait loci (QTL) identification in this species and plant breeding programs focused on phytosanitary Cape gooseberry problems.

### Gene model prediction in *P. Peruviana*

The genome of *P. peruviana* has not been sequenced yet, nevertheless it is possible to generate gene model predictions using the *P. peruviana* transcriptome and the genomes of *Solanum lycopersicum* (tomato) or *Solanum tuberosum* (potato), which are the two closest related species that have genome sequence available [46,47]. The cDNA to genomic DNA alignments were generated using the Splign software package [48] as described in Methods. All the assembled transcripts through the previous steps including 23,964 isotigs and 9,909 singletons, were mapped to the *S. lycopersicum* genome, resulting in 12,436 (36.7%) aligned cDNAs, representing 8,801 gene loci and 9,454 transcript models. On the other hand, 14,515 (42.9%) *P. peruviana* cDNAs were mapped to the *S. tuberosum* genome, representing 10,166 gene loci and 10,992 transcript models, as summarized in Table 6. Splign requires the consensus intron sequences (GT/AG or GC/AG) at the splice sites; therefore strand orientation for the multiple-exon alignments from the Splign output can be decided by the 4-nucleotide sequences at the two intron(s) ends. At the moment, no strand orientation is assigned to single exon transcripts, as the query sequence gets aligned to a continuous region in the genome, unless there is strong polyadenylation signal.

**Table 6:** Cape gooseberry gene model prediction overview from alignments to the tomato and potato genomes

	**Aligned cDNAs**	**Gene loci**	**Transcript models**
*S. lycopersicum* genome	12,436	8,801	9,454
*S. tuberosum* genome	14,515	10,166	10,992

**Figure 4:**
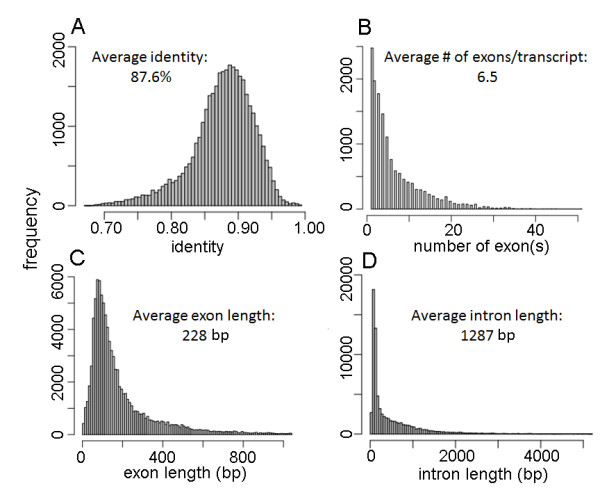
**Predicted gene models features for alignments of *****P. peruviana***** to the* S. tuberosum *genome. A**) Distribution of exon identities. **B**) Distribution of number of exons per transcript. **C**) Distribution of exon lengths. **D**) Distribution of intron lengths.

The majority of aligned exons have an identity with the genomic sequence ranging from 70% to 95%, with an average identity of 87.6%. Figure 4 shows a number of features of the gene models from alignments of *P. peruviana* to *S. tuberosum* genome. Most of gene models contain less than 20 exons. The longest one has 51 exons. The average length of the aligned exons is 228 base pairs and that of the intron is 1,287 base pairs. The intron-exon boundaries as predicted by cDNA to genome alignments are highly conserved when both the *S. tuberosum* and *S. lycopersicum* genomes are used to align the *P. peruviana* transcriptome. We have generated General Feature Format (GFF) files for the gene models ( Additional file 3: ‘Cape gooseberry gene model predictions using the tomato genome’ and Additional file 4: ‘Cape gooseberry gene model predictions using the potato genome’).

**Figure 5:**
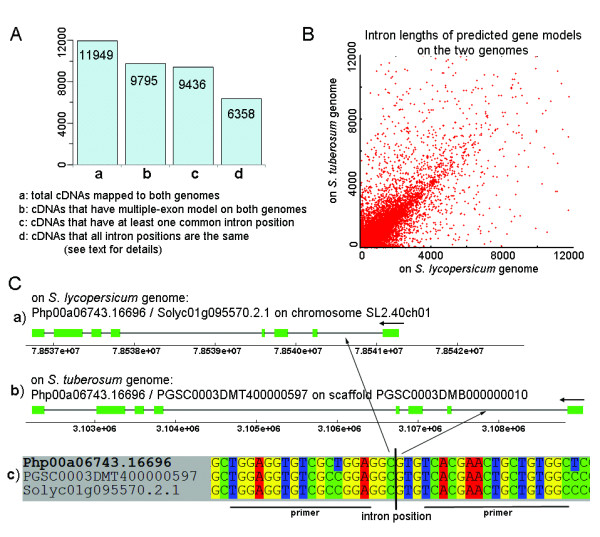
**Gene model prediction in *****P. peruviana**.* A) The number of *P. peruviana* cDNAs that (**a**) can be mapped to both *S. lycopersicum* and *S. tuberosum* genomes; (**b**) have multiple-exon models on both genomes; (**c**) have at least one common intron position (on the cDNA); (**d**) all introns positions are the same in gene models from the two genomes. **B)** For those “common” intron points, the intron lengths in the predicted gene models on the two genomes have big variation. **C)** A typical example: (**a**) *P. peruviana* cDNA Php00a06743.16696 and *S. lycopersicum* cDNA Solyc01g095570.2.1 have identical gene models on *S. lycopersicum* chromosome SL2.40ch01. (**b**) The same *P. peruviana* cDNA and *S. tuberosum* cDNA PGSC0003DMT400000597 have identical gene models on *S. tuberosum* superscaffold PGSC0003DMB000000010. The exon sets of the *P. peruviana* cDNA in panel A and panel B are identical but the two intron sets have remarkable differences. (**c**) Nucleotide sequences at the first intron junction. Primers can be designed at the indicated positions to amplify intron regions from the Solanaceae.

Further examination of the gene models revealed that there are 11,949 *P. peruviana* cDNAs mapped to both *S. lycopersicum* and *S. tuberosum* genome as shown in Figure 5 (panel A-a), among which 9,795 cDNAs have multiple-exon gene models on both genomes (panel A-b). 9,436 (96%) multiple-exon cDNAs have at least one intron occuring at exactly the same position on the cDNA when aligned to the *S. lycopersicum* and *S. tuberosum* genomes (panel A-c). Furthermore, there are 6,358 cDNAs having exactly the same set of intron positions on the cDNA when mapped to the two genomes (Figure 5 panel A-d). However, for those intron positions that have the same coordinates in the cDNA when mapped to the two genomes, the length and thus the sequence of corresponding introns in the gene models from the two genomes have large variances, as revealed in Figure 5 B.

Intron length variation is exemplified in Figure 5C, where a *P. peruviana* cDNA (ID Php00a06743.16696) was mapped to both the *S. lycopersicum* and *S. tuberosum* genomes, resulting in two identical sets of exons, but different sets of intron lengths (a, b). There is also a number of *S. lycopersicum* and *S. tuberosum* cDNAs that have the same predicted gene model in their own genome, respectively (all the cDNAs are aligned by Splign). Figure 5C (c) shows the nucleotide sequences around the first intron site of the 3 cDNAs from *P. peruviana*, *S. tuberosum* and *S. lycopersicum*. Primers targeting conserved flanking exonic regions as indicated can be used to amplify intronic fragments from all three species, *P. peruviana*, *S. lycopersicum* and *S. tuberosum*.

The conserved orthologous set (COS) markers are sets of genes conserved throughout evolution in both sequence and copy number [49,50] that have been used extensively in comparative genomic and phylogenetic studies in *Solanaceae*. The COS marker strategy involves design of universal exonic primers among closely related species based on ortholog identification and multiple sequence alignment to amplify intronic/exonic regions. In the present study, we present another convenient approach to find universal exon regions - gene model prediction by Splign using two or more related genomes to define common models. Given the fact that the *P. peruviana* genome is not available yet, and genomes of both *S. lycopersicum* and *S. tuberosum* are only in their initial versions, gene model predictions would be particularly valuable in obtaining specific intronic regions for marker and SNP discovery in non-model species, as well as for comparative genomic and phylogenetic studies.

We also aligned the *S. lycopersicum* transcriptome to its own genome (data from http://solgenomics.net/organism/solanum_lycopersicum/genome) and also to the *S. tuberosum* genome [48] using Splign. We mapped 34,704 from a total of 34,727 *S. lycopersicum* cDNAs to its own genome with 100% identity (data not shown). However, only 28,366 (81.7%) *S. lycopersicum* cDNAs can be mapped to *S. tuberosum* genome with an average identity of 90%. In the Cape gooseberry case only 42.9% *P. peruviana* cDNAs get mapped to the *S. tuberosum* genome, suggesting that *P. peruviana* is evolutionarily more distant from *S. lycopersicum* and *S. tuberosum* than the two species from each other. We then conducted further analysis to estimate the phylogenetic location of *P. peruviana* in the Solanaceae family.

### Experimental validation of intron positions

The experimental validation of predicted exon/intron boundaries in the assembled cDNAs was carried out in a small sample of cDNAs, which are putative homologous of plant disease resistance genes and can be mapped to both the potato and tomato genomes. For each of these cDNAs, a pair of COSII primers was designed to span one putative intron (based on the computational predicted gene model) for PCR amplification of the genomic DNA. The information of the primers used is summarized in Table 7. All the amplified PCR products had the expected length and then were sequenced using conventional Sanger sequencing. Comparison of the amplified genomic fragments to their corresponding cDNAs revealed that all the eight samples we sequenced indeed showed the exon/intron boundaries consistent with the gene models predicted by Splign. Three of them had the experimentally identified intron positions exactly the same as the predicted. In the other five samples, the predicted intron positions are a few base pairs (1–6 bp) away from the experimentally identified sites. The results are shown in Table 8.

**Table 7:** Primers used for experimental validation of intron positions

**COSII Marker**	**Primer sequences (5′ – 3′)**	***P. peruviana*** **unique identifier**	**Primer position in the cDNA**
C2_At3g07100	ACGAACGATGTGCTGCTGGATATAC	Php00a01046.06900	F-1156/1178
	AGACCCTGGGGATCTAAGCTCTCTG		R-997/1022
C2_At 2 g35920	TGCTTGCAACCAACATAGCTGAG	Php00a05845.15798	F-424/446
	AAGCTCTTGTAGTGGTGTTCGAAG		R-172/195
C2_At 3 g06580	TGCTCAACTCACATGTGAGTGTGAAAG	Php00a06435.16388	F-715/740
	AGCAAACCCAGATTTTGCCATAAC		R-784/806
C2_At 5 g41480	TATTCGTGCTGGTCTGGAGAGTGC	Php00a02812.11168	F-836/859
	ATGATCCTTGTCATTCGCCATAGC		R-646/669
C2_At 5 g27620	ATCTACAATGGTCCGTGATGGAAC	Php00a03329.12202	F-776/799
	TTCCTCTGCCTTGCAAGCTGC		R-720/740
C2_At 3 g04870	ACGCGTGCTAGTATCCAGAGG	Php00a02563.10671	F-1226/1246
	TGACATGGCAAAGCCCACTAACATAC		R-954/977
C2_At 5 g60160	ACACAATGCTAATCAACGTTATGC	Php00a01985.09526	F-278/297
	TCATCCACCGCGCACATTTC		R-482/505

**Table 8:** Comparison of validated exon/intron boundaries between PCR results and the predicted gene models

***P. peruviana unique identifier***	**Position of boundary of the 2 adjacent exons at the mRNA**		**Position of boundary of the 2 adjacent exons at the mRNA**	**Match predicted gene model?**
	**PCR results**	**Predicted model**		
Php00a01046.06900	1077/1078	1077/1078	Precisely
Php00a05845.15798	297/298	294/295	3 bp shift
Php00a02208.09960	653/654	647/648	6 bp shift
Php00a06435.16388	774/775	774/775	Precisely
Php00a02812.11168	751/752	749/750	2 bp shift
Php00a03329.12202	756/757	756/757	Precisely
Php00a02563.10671	1040/1041	1037/1038	3 bp shift
Php00a01985.09526	416/417	417/418	1 bp shift

### Phylogenetic relationship of *P. Peruviana* with other solanaceae species

We found five putative orthologs among *P. peruviana, S. lycopersicum, S. tuberosum, Capsicum spp* (pepper)*, S. melongena* (eggplant) and *Petunia spp*. The proteins are: xyloglucanase inhibitor containing pepsin_retropepsin superfamily domain, mitochondrial catalytic protein containing PP2Cc superfamily domain, mitochondrial small ribosomal subunit protein containing RPS2 superfamily domain, phosphate transporter and a functionally unknown protein.

**Figure 6:**
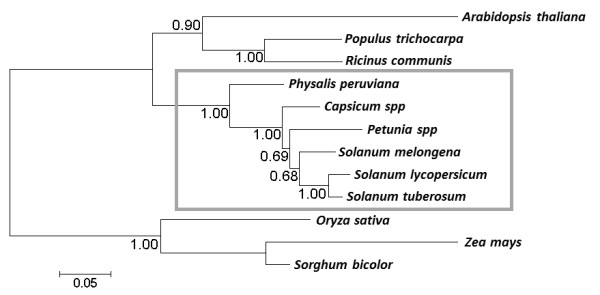
**Phylogenetic relationship among Solanaceae species. **The Cape gooseberry, *P. peruviana* is phylogenetically branched out before the divergence of the other five Solanaceae family members: *S. lycopersicum*, *S. tuberosum*, *Capsicum spp*, *S. melongena* and *Petunia spp*. The numbers are bootstrapping values. The tree was constructed using the amino acid substitution model with PhyML package.

To obtain the best accuracy of the phylogenetic tree to be built, we compared the five putative orthologous proteins to the NCBI’s plant RefSeq protein database. There are seven other species that have BLAST hits with an expect value < 1e-5 to all the five orthologs from the previous steps. These seven species are: *Arabidopsis thalianaPopulus trichocarpa* (black cottonwood), *Ricinus communis* (castor bean), *Vitis vinifera* (grape), *Oryza sativa* (rice), *Zea mays* (corn) and *Sorghum bicolor*, none of which belongs to the Solanaceae family. The phylogenetic tree was constructed between thirteen plants using the software Phyml [51] and MEGA [52]. The tree generated has good bootstrapping support at all of the branch points except for the position of *V. vinifera*. We removed the *V. vinifera* sequence and constructed the tree presented in Figure 6.

The phylogenetic relationship among *S. lycopersicumS. tuberosumCapsicum sppS. melongena* and *Petunia spp* is consistent with a previous study by Wang Y et al. [53], in which the tree was constructed based on an unduplicated conserved syntenic segment in the genomes of the five plants. Our results showed that *P. peruviana* branched out before the divergence of the other five Solanaceae family members. Details of the phylogenetic analysis are summarized in Additional file 5: ‘Phylogenetic analysis workflow’.

## Conclusions

This report constitutes the first genomic resource for the *Physalis* genus providing a large collection of assembled and functionally annotated cDNAs. The *Physalis* genus is part of the Solanaceae family, whose members are important sources of food, spice and medicine. However, genomic data for other members of the *Physalis* genus is limited. Therefore, this resource will enhance comparative studies within the family and the transcriptome will serve as a starting point for gene discovery in *Physalis* and for future annotations of the *Physalis peruviana* genome sequence. A number of the genes identified in this study provide candidates for resistance genes against viruses, fungal or bacterial pathogens. Additionally, this study is a potentially invaluable resource for mapping and marker-assisted breeding in *Physalis peruviana* and closely related species like *Physalis philadelphica*, commonly known as tomatillo, which are food staples in Central American countries.

## Methods

### cDNA synthesis and cDNA library normalization

Fresh leaf tissue from the Cape gooseberry *Physalis peruviana* Colombian ecotype plants from the Colombian *in vitro* germplasm bank (accession number 09U216-6) at the Corporacion Colombiana de Investigacion Agropecuaria (CORPOICA) were processed and flash frozen in liquid nitrogen. Tissues were immediately sent to Bio S&T Inc. (Montreal, QC, Canada) where RNA extraction, cDNA synthesis and normalization were performed. Briefly, RNA was extracted using a modified TRIzol method (Invitrogen, USA). cDNA synthesis was carried out using 16 μg total RNA by a modified SMART™ cDNA synthesis method and then were normalized by a modified normalization method [54,55] where full-length cDNA was synthesized with two set of primers for driver and tester cDNA. Single-stranded cDNA was used for hybridization instead of double-stranded cDNA. Excess amounts of sense-stranded cDNA hybridized with antisense-stranded cDNA. After hybridization, duplex DNA was removed by hydroxyapatite chromatography. Normalized tester cDNA was re-amplified and purified with tester specific primer L4N by failsafe^Tm^ PCR (Epicentre Biotechnologies, USA), while driver cDNA was unable to amplify using L4N primer. Size fractionation of re-amplified cDNA was done in a 1% agarose gel. Greater than 0.5 kb cDNA fragments were purified by electroelution and after determining the concentrations, purified cDNAs were precipitated and stored in 80% EtOH at −80°C.

### cDNA sequencing and assembly

The normalized cDNA library was prepared for sequencing at Emory Genomics Center (Atlanta, GA, USA). Approximately 5 μg of purified cDNA was sheared into small fragments via Covaris E210 Acoustic Focusing Instrument and sequenced in three-fourths 454 plate run on a 454 GS-FLX Titanium platform (Roche). The SFF files containing raw sequences and quality scores were submitted to the NCBI Sequence Read Archive (accession number SRP005904).

SeqClean [19,56] was used before and after the assembly, for automated trimming and validation of the raw read files and the assembled file. SeqClean was launched with a minimum and maximum length cut-off of 50pb and 600pb. We used the Newbler software, GS *de novo* Assembler (Roche, version 2.5.3) with default parameters, to assemble reads into contigs, then further into isotigs. Isotigs within an isogroup represent putative alternatively spliced transcripts of a gene. Reads that cannot be connected with any others were defined as singletons.

### Functional annotation

After assembly, a local BLASTX [22,23] was used to compare the assembled isotigs and singletons against the UniProtKB/Swiss-Prot database (released on April-2011) using an expect value threshold of 1e-5. The remaining cDNAs that did not get hits from UniProtKB/Swiss-Prot were compared against the NCBI RefSeq database (Release 47). The BLASTX output (XML format) was subjected to Blast2GO [24] for Gene Ontology (GO) analysis. Blast2GO retrieves the most significant GO terms associated with the obtained hits to the query sequence. When possible, Blast2GO also provides Enzyme commission (EC) numbers and the metabolic pathways they participate. We also compared all the *P. peruviana* cDNAs against the NCBI CDD database [30] using an expect value threshold of 1e-5 and selected all the hits where the aligned length is more than 2/3 of the targeted CDD length for domain identification.

### SSR identification

Phobos (version 3.3.11) (http://www.rub.de/spezzoo/cm/cm_phobos.htm) was used to identify microsatellites (SSRs) in the publicly available collection of assembled transcripts and singletons [GenBank: JO124085-JO157957]. Perfect and imperfect searches were performed using default parameters.

### Gene model prediction

Gene model prediction was carried out using the software package Splign [48], which has been proven to be able to accurately compute cDNA-to-Genome alignment with high efficiency. At the heart of the program is a compartmentization algorithm which identifies possible gene duplications, and a refined alignment algorithm recognizing introns and splice signals. The complete genome of *P. peruviana* is not available yet. The two closest relatives of *P. peruviana* that have genomic sequences available are *S. lycopersicum* (tomato) (data from http://solgenomics.net/organism/solanum_lycopersicum/genome; ITAG Release 2.3) and *S. tuberosum* (potato; PGSC_DM_v3.4) [47]. Therefore we used the draft genomes of potato and tomato as the reference genome to map the assembled *P. peruviana* leaf transcriptome.

### Phylogenetic analysis

We selected orthologous proteins using the all-to-all alignment and mutual best hits selection strategy [57]. Pairwise alignments were performed using BLASTP (expect value < 1e-5) using the RefSeq proteins from *S. lycopersicumS. tuberosumCapsicum sppS. melongena* and *Petunia spp*. At the time of analysis, the numbers of RefSeq proteins from the five species were: 4,788 for *S. tuberosum*; 6,008 for *S. lycopersicum*; 263 for *S. melongena*; 1,701 for *Capsicum spp* and 1,226 for *Petunia spp*. Fifteen putative orthologous proteins were found, which are present in all five species. Next, we aligned the assembled *P. peruviana* isotigs using BLASTX (expect value < 1e-5) against the database made of the fifteen orthologous groups obtained from the previous step (altogether 75 proteins). We identified eleven orthologous groups of proteins from all the five plants with hit(s) from the *P. peruviana* transcriptome. The best hit was chosen when multiple *P. peruviana* proteins hit a given group. We manually examined the alignments in eleven clusters and removed those with large length variation (the longest one is >20% of the shortest one) and susceptible similarities (< 65%). Thereafter we ended up with five orthologous groups among the six species.

To obtain higher accuracy phylogenetic tree, we further compared the five orthologous groups against the entire plant RefSeq protein database using BLASTP. There are altogether seven more plants that have significant hit(s) (expect value < 1e-5) for all the five orthologous groups. To this step we have thirteen plants for the five orthologous groups. We concatenated the five proteins in each species (in the same order) and aligned them using the program MUSCLE [58]. The alignment results were manually refined and subjected to Phyml [51] and MEGA version 5 [52] for phylogenetic tree construction. Bootstrapping was repeated 1,000 times. Both programs produced the same results.

## Misc

Gina A. Garzón-Martínez and Z. Iris Zhu contributed equally to this work.

## Competing interests

The authors declare that they have no competing interests.

## Authors’ contributions

GG-M acquired the sequencing data, performed the genome assembly, the functional annotation, SSR marker identification and drafted the manuscript. ZIZ participated in the genome assembly, performed functional annotation, gene model prediction, phylogenetic analysis and drafted the manuscript. DL participated in the design of the study and contributed to the phylogenetic analysis. LSB conceived of the study, and participated in its design and coordination and helped to draft the manuscript. LM-R conceived of the study, and participated in its design and coordination and helped to draft the manuscript. All authors read and approved the final manuscript.

## Supplementary Material

Additional file 1: ** Cape gooseberry cDNAs.** The annotated FASTA sequences of the assembled transcriptome, including singletons. Click here for file

Additional file 2: ** Functional annotation of ten *****Physalis peruviana***** SSRs markers related to plant defense.** Click here for file

Additional file 3: ** Cape gooseberry gene model predictions using the tomato genome.** Gene model predictions using Splign from cDNA to genome alignments to the tomato genome. Click here for file

Additional file 4: ** Cape gooseberry gene model predictions using the potato genome.** Gene model predictions using Splign from cDNA to genome alignments to the potato genome. Click here for file

Additional file 5: Phylogenetic analysis workflow. Click here for file
