# Supplementary material for: The Physalis peruviana leaf transcriptome: assembly, annotation and gene model prediction
Source: BMC Genomics. 2012 Apr 25;13:151. doi: 10.1186/1471-2164-13-151 (PMC3488962; doi:10.1186/1471-2164-13-151)
Supplement: Additional file 2: — Functional annotation of ten Physalis peruviana SSRs markers related to plant defense. [file 1471-2164-13-151-S2.doc]

**Supplemental Table 1.** Functional annotation of ten *Physalis peruviana* SSRs markers related to plant defense

| **SSR Marker** | **Class** | **Motif** | **Repeat type** | **Start** | **End** | **NCBI ID** | **GO Category ID** | **Functional Annotation** |
| --- | --- | --- | --- | --- | --- | --- | --- | --- |
| SSR1 | Imperfect | Hexanucleotide | ACAGAG | 273 | 291 | gi|340818698|gb|JO126774.1| | C:0005737 | cytoplasm |
|  |  |  |  |  |  |  | P:0010197 | polar nucleus fusion |
|  |  |  |  |  |  |  | P:0009610 | response to symbiotic fungus |
|  |  |  |  |  |  |  | P:0009737 | response to abscisic acid stimulus |
|  |  |  |  |  |  |  | F:0003700 | transcription factor activity |
|  |  |  |  |  |  |  | C:0005634 | nucleus |
|  |  |  |  |  |  |  | F:0046982 | protein heterodimerization activity |
|  |  |  |  |  |  |  | P:0010201 | response to continuous far red light stimulus by the high-irradiance response system |
|  |  |  |  |  |  |  | F:0042803 | protein homodimerization activity |
| SSR2 | Imperfect | Tetranucleotide | AAAG | 300 | 321 | gi|340819396|gb|JO127472.1| | P:0009626 | plant-type hypersensitive response |
|  |  |  |  |  |  |  | P:0010204 | defense response signaling pathway, resistance gene-independent |
|  |  |  |  |  |  |  | F:0016301 | kinase activity |
|  |  |  |  |  |  |  | P:0002764 | immune response-regulating signaling pathway |
|  |  |  |  |  |  |  | P:0016045 | detection of bacterium |
| SSR3 | Imperfect | Trinucleotide | AAG | 80 | 97 | gi|340821154|gb|JO129230.1| | P:0009626 | plant-type hypersensitive response |
|  |  |  |  |  |  |  | F:0004872 | receptor activity |
|  |  |  |  |  |  |  | P:0006952 | defense response |
|  |  |  |  |  |  |  | P:0033554 | cellular response to stress |
|  |  |  |  |  |  |  | P:0023060 | signal transmission |
|  |  |  |  |  |  |  | P:0012501 | programmed cell death |
|  |  |  |  |  |  |  | C:0044425 | membrane part |
|  |  |  |  |  |  |  | F:0032559 | adenyl ribonucleotide binding |
|  |  |  |  |  |  |  | C:0044424 | intracellular part |
| SSR4 | Imperfect | Hexanucleotide | AATGGG | 285 | 302 | gi|340822742|gb|JO130818.1| | P:0042538 | hyperosmotic salinity response |
|  |  |  |  |  |  |  | P:0042742 | defense response to bacterium |
|  |  |  |  |  |  |  | P:0080052 | response to histidine |
|  |  |  |  |  |  |  | P:0042939 | tripeptide transport |
|  |  |  |  |  |  |  | P:0009753 | response to jasmonic acid stimulus |
|  |  |  |  |  |  |  | F:0042936 | dipeptide transporter activity |
|  |  |  |  |  |  |  | P:0043201 | response to leucine |
|  |  |  |  |  |  |  | P:0009611 | response to wounding |
|  |  |  |  |  |  |  | P:0009751 | response to salicylic acid stimulus |
|  |  |  |  |  |  |  | P:0009737 | response to abscisic acid stimulus |
|  |  |  |  |  |  |  | F:0042937 | tripeptide transporter activity |
|  |  |  |  |  |  |  | C:0016020 | membrane |
|  |  |  |  |  |  |  | P:0080053 | response to phenylalanine |
|  |  |  |  |  |  |  | P:0042938 | dipeptide transport |
| SSR5 | Imperfect | Hexanucleotide | AAGCTC | 689 | 705 | gi|340823750|gb|JO131826.1| | P:0009409 | response to cold |
|  |  |  |  |  |  |  | P:0045449 | regulation of transcription |
|  |  |  |  |  |  |  | P:0010200 | response to chitin |
|  |  |  |  |  |  |  | P:0050832 | defense response to fungus |
| SSR6 | Imperfect | Hexanucleotide | AGGGGG | 1424 | 1464 | gi|340824981|gb|JO133057.1| | P:0009789 | positive regulation of abscisic acid mediated signaling pathway |
|  |  |  |  |  |  |  | P:0006979 | response to oxidative stress |
|  |  |  |  |  |  |  | P:0052544 | callose deposition in cell wall during defense response |
|  |  |  |  |  |  |  | P:0009753 | response to jasmonic acid stimulus |
|  |  |  |  |  |  |  | P:0031348 | negative regulation of defense response |
|  |  |  |  |  |  |  | P:0009651 | response to salt stress |
|  |  |  |  |  |  |  | P:0042742 | defense response to bacterium |
|  |  |  |  |  |  |  | P:0009926 | auxin polar transport |
|  |  |  |  |  |  |  | P:0010119 | regulation of stomatal movement |
|  |  |  |  |  |  |  | P:0009408 | response to heat |
|  |  |  |  |  |  |  | F:0005515 | protein binding |
|  |  |  |  |  |  |  | P:0010150 | leaf senescence |
|  |  |  |  |  |  |  | P:0048765 | root hair cell differentiation |
|  |  |  |  |  |  |  | P:0009871 | jasmonic acid and ethylene-dependent systemic resistance, ethylene mediated signaling pathway |
|  |  |  |  |  |  |  | P:0001736 | establishment of planar polarity |
|  |  |  |  |  |  |  | P:0050832 | defense response to fungus |
|  |  |  |  |  |  |  | P:0010182 | sugar mediated signaling pathway |
| SSR7 | Perfect | Trinucleotide | AAC | 263 | 285 | gi|340828480|gb|JO136556.1| | P:0042742 | defense response to bacterium |
|  |  |  |  |  |  |  | P:0006355 | regulation of transcription, DNA-dependent |
|  |  |  |  |  |  |  | P:0009611 | response to wounding |
|  |  |  |  |  |  |  | P:0009751 | response to salicylic acid stimulus |
|  |  |  |  |  |  |  | P:0050691 | regulation of defense response to virus by host |
|  |  |  |  |  |  |  | P:0010200 | response to chitin |
|  |  |  |  |  |  |  | F:0005515 | protein binding |
|  |  |  |  |  |  |  | F:0003700 | transcription factor activity |
|  |  |  |  |  |  |  | P:0050832 | defense response to fungus |
|  |  |  |  |  |  |  | C:0005634 | nucleus |
| SSR8 | Imperfect | Hexanucleotide | AAAAAT | 33 | 52 | gi|340830384|gb|JO138460.1| | P:0052544 | callose deposition in cell wall during defense response |
|  |  |  |  |  |  |  | P:0031348 | negative regulation of defense response |
|  |  |  |  |  |  |  | C:0005739 | mitochondrion |
|  |  |  |  |  |  |  | P:0006855 | drug transmembrane transport |
|  |  |  |  |  |  |  | F:0042626 | ATPase activity, coupled to transmembrane movement of substances |
|  |  |  |  |  |  |  | P:0042742 | defense response to bacterium |
|  |  |  |  |  |  |  | P:0015691 | cadmium ion transport |
|  |  |  |  |  |  |  | C:0009941 | chloroplast envelope |
|  |  |  |  |  |  |  | P:0009627 | systemic acquired resistance |
|  |  |  |  |  |  |  | P:0009817 | defense response to fungus, incompatible interaction |
|  |  |  |  |  |  |  | P:0042344 | indole glucosinolate catabolic process |
|  |  |  |  |  |  |  | F:0015086 | cadmium ion transmembrane transporter activity |
|  |  |  |  |  |  |  | C:0005886 | plasma membrane |
| SSR9 | Imperfect | Pentanucleotide | AGATG | 1717 | 1734 | gi|340831495|gb|JO139571.1| | P:0042742 | defense response to bacterium |
|  |  |  |  |  |  |  | P:0045449 | regulation of transcription |
|  |  |  |  |  |  |  | F:0005515 | protein binding |
|  |  |  |  |  |  |  | F:0003700 | transcription factor activity |
|  |  |  |  |  |  |  | P:0010182 | sugar mediated signaling pathway |
|  |  |  |  |  |  |  | C:0005634 | nucleus |
|  |  |  |  |  |  |  | P:0009873 | ethylene mediated signaling pathway |
| SSR10 | Imperfect | Hexanucleotide | ACGAGT | 753 | 768 | gi|340834280|gb|JO142356.1| | P:0052544 | callose deposition in cell wall during defense response |
|  |  |  |  |  |  |  | P:0042742 | defense response to bacterium |
|  |  |  |  |  |  |  | P:0006075 | 1,3-beta-glucan biosynthetic process |
|  |  |  |  |  |  |  | F:0003843 | 1,3-beta-glucan synthase activity |
|  |  |  |  |  |  |  | P:0009965 | leaf morphogenesis |
|  |  |  |  |  |  |  | P:0000003 | reproduction |
|  |  |  |  |  |  |  | P:0009870 | defense response signaling pathway, resistance gene-dependent |
|  |  |  |  |  |  |  | P:0009555 | pollen development |
|  |  |  |  |  |  |  | P:0009863 | salicylic acid mediated signaling pathway |
|  |  |  |  |  |  |  | C:0000148 | 1,3-beta-glucan synthase complex |
|  |  |  |  |  |  |  | P:0010150 | leaf senescence |
|  |  |  |  |  |  |  | P:0050832 | defense response to fungus |
