# Supplementary figures and images for: The Physalis peruviana leaf transcriptome: assembly, annotation and gene model prediction
Source: BMC Genomics. 2012 Apr 25;13:151. doi: 10.1186/1471-2164-13-151 (PMC3488962; doi:10.1186/1471-2164-13-151)

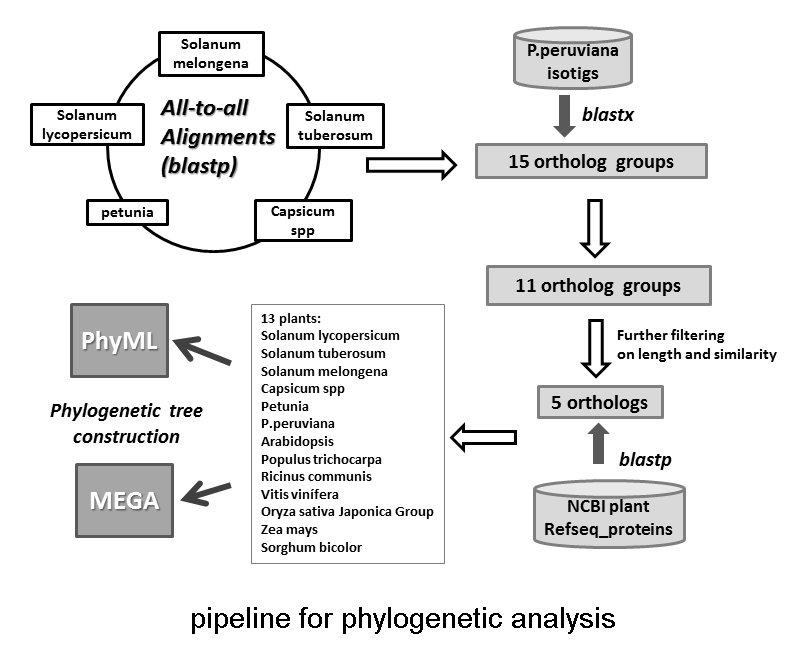

Supplement: Additional file 5: — Phylogenetic analysis workflow. [file 1471-2164-13-151-S5.tiff]
